# Aphakia treatment with sutureless scleral fixation or retropupillary iris-claw intraocular lens implantation: visual acuity, anterior segment and keratometry outcomes

**DOI:** 10.1007/s10792-024-03187-6

**Published:** 2024-06-24

**Authors:** Müslüm Toptan, Omer Faruk Yilmaz, Halit Oguz

**Affiliations:** 1 Department of Ophthalmology, School of Medicine, Harran University, Sanlıurfa, Turkey; 2 Department of Ophthalmology, Göztepe Training and Research Hospital, Istanbul, Turkey; 3 Department of Ophthalmology, Medeniyet University Göztepe Prof. Dr. Süleyman Yalçin City Hospital, Istanbul, Turkey

**Keywords:** Aphakia, Iris claw, Sutureless scleral fixation, Modified Yamane technique, Anterior segment structures, Scheimpflug corneal topography

## Abstract

**Purpose:**

Evaluation of anterior segment parameters using the Scheimpflug corneal topography 1 year after surgery in patients who underwent sutureless scleral fixation intraocular lens (SFIOL) implantation using the modified Yamane technique and retropupillary iris-claw intraocular lens (RPIOL) implantation.

**Methods:**

A total of 60 eyes from 57 patients who underwent sutureless SFIOL implantation and 57 eyes from 52 patients who underwent RPIOL implantation were included. Anterior chamber depth (ACD), anterior chamber angle (ACA), anterior chamber volume (ACV), anterior–posterior corneal astigmatism, and keratometric values were assessed using the Scheimpflug corneal topography (Pentacam HR, Germany).

**Results:**

There was no statistically significant difference in postoperative UCVA and BCVA between the sutureless SFIOL and the RPIOL group (*p* = 0.236, *p* = 0.293, respectively). While there was no statistically significant difference in postoperative IOP between the two groups (*p* = 0.223), a statistically significant decrease in IOP was observed in both groups (*p* < 0.001). While there was no statistical difference between the sutureless SFIOL group and the RPIOL group in terms of spherical value (*p* = 0.441) and spherical equivalence (*p* = 0.237), there was a statistically significant difference in cylindrical value (*p* < 0.001). While there was a statistical difference in anterior astigmatism (*p* < 0.001), there was no statistical difference in posterior astigmatism (*p* = 0.405). There was no statistical difference in terms of ACV, ACD, and ACA between the sutureless SFIOL and the RPIOL group (*p* = 0.812, *p* = 0.770, *p* = 0.401, respectively).

**Conclusion:**

In this study, although there was a statistical difference in cylindrical value and anterior corneal astigmatism between the sutureless SFIOL and RPIOL groups, vision was not affected. According to this study, sutureless SFIOL and RPIOL are two successful methods in terms of visual acuity, anterior segment, and keratometry outcomes in aphakic patients after phacoemulsification.

## Introduction

In secondary lens implantation surgery for aphakic patients, various complications may arise depending on factors such as the type of lens utilized, surgical technique employed, and the condition of the cornea [1]. This scenario has prompted surgeons to explore new intraocular lenses (IOL) and anatomically appropriate surgeries characterized by shorter durations and lower complication rates [2]. Scleral fixation of 3-piece IOL using non-absorbable sutures has traditionally been the accepted technique for IOL placements [3]. However, studies comparing sutured and sutureless IOL have shown that both methods are equally effective and provide excellent visual results even after 2 years of follow-up [4]. Sutureless scleral fixation intraocular lens (SFIOL) implantation using the Yamane technique has also been widely accepted as a minimally invasive procedure [5]. In recent years, there has been a growing interest retropupillary iris-claw intraocular lens (RPIOL) due to the ease of surgery, simplicity of retropupillary application, high anatomical suitability, and minimal endothelial damage [6].

Some studies have indicated that phacoemulsification leads to increases in anterior chamber depth (ACD), anterior chamber angle (ACA), and anterior chamber volume (ACV), as well as decreases in IOP, irrespective of the type of IOL implantation [7, 8]. On the contrary, it has been reported that an increase in IOP can be observed after sutureless SFIOL and RPIOL implantation in various studies [9, 10]. Among the IOL commonly used in aphakic patients, there is limited data regarding anatomical suitability and the extent of changes they cause in anterior segment structures. The rising popularity of RPIOL fixation has prompted us to compare the outcomes of this fixation with those of less invasive scleral fixation techniques. By utilizing the Scheimpflug corneal topography, quantitative values with high reproducibility can be obtained regarding anterior segment structures [11]. Our study aims to evaluate anterior segment parameters 1 year after surgery using the Scheimpflug corneal topography in eyes with sutureless SFIOL and RPIOL.

## Methods

This study comprised 109 patients who were admitted to the Ophthalmology Department outpatient clinic of Harran University Faculty of Medicine with a diagnosis of postoperative aphakia. The records of 60 eyes from 57 patients who underwent sutureless SFIOL implantation using the Yamane technique and 57 eyes from 52 patients who underwent RPIOL implantation between July 2017 and June 2023 were retrospectively reviewed. Patients who underwent senile cataract surgery and were left aphakic, and subsequently received either sutureless SFIOL or RPIOL implantation, and were followed for at least 1 year, were included in the study. Both surgical methods were performed by the same surgeon (MT). The decision regarding the choice of IOL implantation was made at the discretion of the operating surgeon. There was no condition that would prevent the placement of an iris claw İOL.

**Fig. 1:**
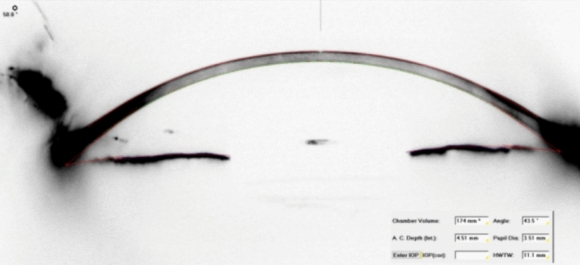
Visualization of the anterior segment structures with the Scheimpflug camera a Image of a patient with Iris-claw IOL implantation

**Fig. 2:**
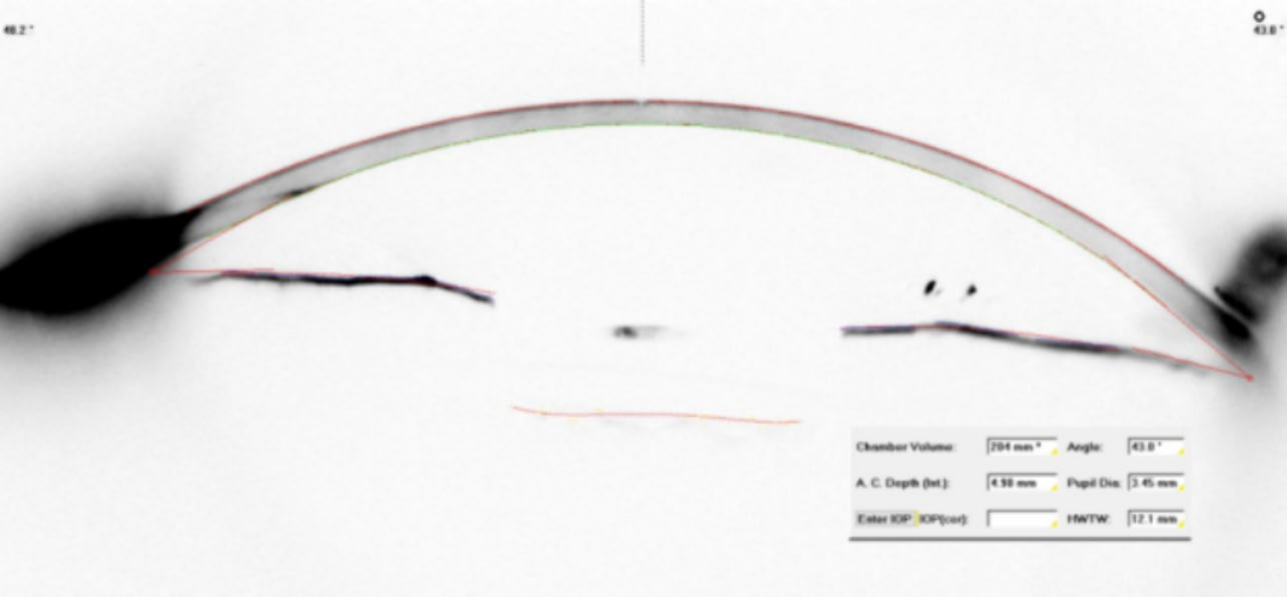
Visualization of the anterior segment structures with the Scheimpflug camera a Image of a patient with sutureless SFIOL implantation

A comprehensive anterior segment and fundus examination was conducted both before and 1 year after surgery. UCVA and BCVA were measured and converted to the logarithm of the minimum angle of resolution (LogMAR). IOP was assessed using Goldman applanation tonometry. Measurements of spherical value, cylindrical value, and spherical equivalent were obtained using an automatic auto-refractometry device (Full Auto Ref-Keratometer, Nidek Co., Ltd., Tokyo, Japan). Anterior segment structures, anterior–posterior corneal astigmatism, and keratometric values were measured using the Scheimpflug corneal topography (Pentacam HR; Oculus Optikgeräte GmbH, Wetzlar, Germany) (Figs. 1 and 2). ACA, ACV, and ACD measurements obtained automatically by the software were calculated from 25 images. Additionally, values and images were recorded from the temporal incision site and nasal, superior, and inferior quadrants in ACA measurements. An optical biometry device (Lenstar LS 900, Haag-Streit, Switzerland) and the SRK/T formula were utilized to calculate IOL power targeting emmetropia and axial length before surgery.

### Exclusion criteria

Patients with glaucoma, retinopathy, vision-impairing corneal scarring, history of penetrating keratoplasty, eyes left aphakic due to trauma, and those who had undergone vitreoretinal surgery other than pars plana vitrectomy were excluded from the study.

### *Iris*-claw IOL implantation

First, peribulbar anesthesia was administered. Then, the conjunctiva was opened, and a 5.5-mm superior corneoscleral tunnel incision was made. Subsequently, triamcinolone acetonide was injected into the anterior chamber, and if necessary, a 23-gauge anterior vitrectomy was performed**.** Pupillary constriction was achieved using pilocarpine. Following this, two limbal paracenteses were performed 180º apart. Subsequently, the iris-claw IOL (Verisyse VRSA54; Abbott Laboratories, Inc., Abbott Park, IL, USA) was positioned over the iris. A haptic was carefully guided below the iris and enclaved to the peripheral iris claw with the assistance of a cannula. The same procedure was repeated for the other haptic. Subsequently, a prophylactic iridectomy was performed. The corneoscleral incision was closed with a single non-tight crossed 10.0 nylon suture, and the wound integrity was verified. Closure of the conjunctiva was accomplished using 8.0 vicryl sutures. The corneoscleral suture was removed two months later.

### Sutureless SFIOL implantation

A temporal clear corneal incision measuring 2.8 mm was created. Following this, triamcinolone acetonide was injected into the anterior chamber, and if deemed necessary, a 23-gauge anterior vitrectomy was performed. A three-piece, foldable, hydrophobic acrylic IOL (Sensar; Advanced Medical Optics, Inc., Santa Ana, CA, USA) was inserted into the anterior chamber. The IOL haptics were externalized via transconjunctival scleral tunnels at the 6 and 12 o’clock positions using a 27 G needle. These transconjunctival scleral tunnels were prepared to match the haptic position and curvature. They were located 2 mm away from the limbus, had a length of 2 mm in the sclera, and were oriented towards the posterior chamber. The tips of the haptics were cauterized to form a terminal knob.

### Statistical analysis

All statistical analyses were performed using IBM SPSS Statistics for Windows, Version 25.0 (IBM Corp., Armonk, NY, USA). Normality of variables was assessed using histogram and Q-Q plots. Data were summarized as mean ± standard deviation or median (1st quartile–3rd quartile) for continuous variables, depending on their distribution, and as frequency (percentage) for categorical variables. Normally distributed variables were compared using independent samples t-test, while variables not following normal distribution were analyzed using the Mann–Whitney U test. Chi-square tests were used for categorical variables. Preoperative versus postoperative comparisons of UCVA and IOP were made using the Wilcoxon signed-rank test. Statistical significance was considered at *p* < 0.05.

## Results

The mean age of the 32 males and 28 females who received sutureless SFIOL was 67.75 ± 5.26 years, whereas the 28 females and 29 males who underwent RPIOL implantation was 69.02 ± 6.6 years. No statistically significant difference was observed in terms of age and gender distribution between the groups that received sutureless SFIOL and RPIOL (*p* = 0.252, *p* = 0.649, respectively).

**Fig. 3:**
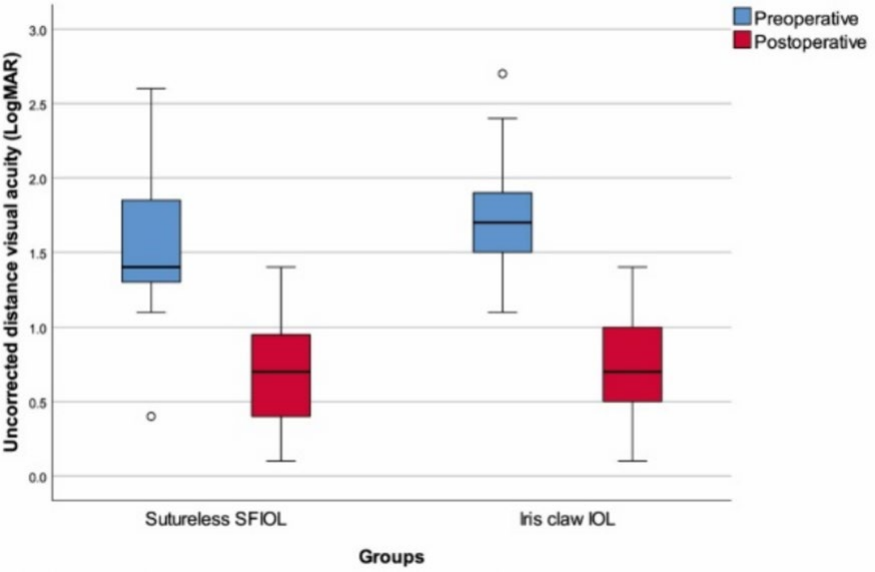
Preoperative and postoperative UCVA with regard to groups

The preoperative UCVA was measured to be 1.6 (1.3–1.85) in the sutureless SFIOL group, while it measured 1.7 (1.5–1.9) in the RPIOL group. There is no statistically significant difference in terms of preoperative UCVA between the sutureless SFIOL group and the RPIOL group (*p* = 0.253). The postoperative UCVA was measured to be 0.6 (0.4–0.95) in the sutureless SFIOL group and 0.6 (0.5–1.0) in the RPIOL group (Fig. 3). There is no statistically significant difference in terms of postoperative UCVA between the sutureless SFIOL and the RPIOL group (*p* = 0.379).

The preoperative BCVA was recorded as 0.8 (0.2–1.5) in the sutureless SFIOL and 0.8 (0.2–1.6) in the RPIOL group. There is no statistically significant difference in terms of preoperative BCVA between the sutureless SFIOL and the RPIOL group (*p* = 0.980). The postoperative BCVA was 0.4 (0.2–0.7) in the sutureless SFIOL and 0.3 (0.2–0.5) in the RPIOL group. There is no statistically significant difference in terms of postoperative BCVA between the sutureless SFIOL and the RPIOL group (*p* = 0.193).

The preoperative IOP was 18 (16.5–19) mmHg in the sutureless SFIOL group and 18 (18–19) mmHg in the RPIOL group. There is no statistically significant difference in terms of preoperative IOP between the sutureless SFIOL and the RPIOL group (*p* = 0.265). The postoperative IOP was 16 (15–18) mmHg in the sutureless SFIOL group and 16 (15–18) mmHg in the RPIOL group. There is no statistically significant difference in terms of postoperative IOP between the sutureless SFIOL and the RPIOL group (*p* = 0.223). The reduction in IOP was − 2 (− 5–0) mmHg in the sutureless SFIOL and − 2 (− 4–0) mmHg in the RPIOL group. A statistically significant decrease in IOP was observed in both the sutureless SFIOL and the RPIOL group (*p* < 0.001).

In the sutureless SFIOL group, the spherical value was − 0.50 D (− 0.75–− 0.23), the cylindrical value was − 1.25 D (− 1.50–− 0.75), and the spherical equivalent value was − 0.74 ± 0.42. In the RPIOL group, the spherical value was − 0.50 (− 0.75–− 0.25), the cylindrical value was − 1.50 (− 1.75–− 1.25), and the spherical equivalent value was − 0.83 ± 0.47. While there is no statistical difference between the sutureless SFIOL and the RPIOL group in terms of spherical value (*p* = 0.441) and spherical equivalence (*p* = 0.237), there is a statistically significant difference in cylindrical value (*p* < 0.001).

In the sutureless SFIOL group, the ACV was 186.40 ± 30.82 mm^3^, the ACD was 3.72 ± 0.58 mm, and the ACA was 40.69° ± 5.01°. In the RPIOL group, the ACV was 185.00 ± 32.57 mm^3^, the ACD was 3.69 ± 0.58 mm, and the ACA was 41.47° ± 5.03°. There is no statistical difference in terms of ACV, ACD, and ACA between the sutureless SFIOL group and the RPIOL group (*p* = 0.812, *p* = 0.770, *p* = 0.401, respectively).

In the sutureless SFIOL group, the keratometry readings were K1: 42.47 ± 0.63 D and K2: 43.83 ± 1.05 D, while in the RPIOL group, they were K1: 42.56 ± 0.75 D and K2: 44.02 ± 1.21 D. There was no statistical difference observed between the sutureless SFIOL and the RPIOL group in terms of K1 (*p* = 0.513) and K2 (*p* = 0.349). In the sutureless SFIOL group, the anterior astigmatism was 0.9 D (0.7–1.4), and the posterior astigmatism was 0.2 D (0.2–0.3). In the RPIOL group, the anterior astigmatism was 1.5 D (0.9–1.8), and the posterior astigmatism was 0.3 D (0.2–0.3). While there was a statistical difference in anterior astigmatism (*p* < 0.001), there was no statistical difference in posterior astigmatism (*p* = 0.405) (Table 1).

**Table 1:** Summary of patients characteristics and measurements with regard to groups

	Sutureless SFIOL (n = 60)	Iris claw IOL (n = 57)	*p*
Age (years)	67.75 ± 5.26	69.02 ± 6.60	0.252
Sex			
Female	32 (53.3%)	28 (49.1%)	0.649
Male	28 (46.7%)	29 (50.9%)	
Uncorrected distance visual acuity (LogMAR)			
Preoperative	1.6 (1.3–1.85)	1.7 (1.5–1.9)	0.253
Postoperative	0.6 (0.4–0.95)	0.6 (0.5–1.0)	0.379
*p* (within groups)	**< 0.001**	**< 0.001**	
Change^a^	− 1.0 (− 1.3–− 0.4)	− 1.1 (− 1.3–− 0.7)	0.236
Best corrected distance visual acuity (LogMAR)			
Preoperative	0.8 (0.2–1.5)	0.8 (0.2–1.6)	0.980
Postoperative	0.4 (0.2–0.7)	0.3 (0.2–0.5)	0.193
* p* (within groups)	**< 0.001**	**< 0.001**	
Change^a^	− 0.4 (− 0.8–− 0.2)	− 0.5 (1.3–− 0.2)	0.293
Intraocular pressure (mmHg)			
Preoperative	18 (16.5–19)	18 (18–19)	0.265
Postoperative	16 (15–18)	16 (15–18)	0.223
Change^a^	− 2 (− 5–0)	− 2 (− 4–0)	**< 0.001**
Spherical value, postoperative (D)	− 0.50 (− 0.75–− 0.23)	− 0.50 (− 0.75–− 0.25)	0.441
Cylindrical value, postoperative (D)	− 1.25 (− 1.50–− 0.75)	− 1.50 (− 1.75–− 1.25)	**< 0.001**
Spherical equivalent, postoperative (D)	− 0.74 ± 0.42	− 0.83 ± 0.47	0.237
Anterior chamber volume, postoperative (mm^3^)	186.40 ± 30.82	185.00 ± 32.57	0.812
Anterior chamber depth, postoperative (mm)	3.72 ± 0.58	3.69 ± 0.58	0.770
Anterior chamber angle, postoperative (°)	40.69 ± 5.01	41.47 ± 5.03	0.401
K1, postoperative (D)	42.47 ± 0.63	42.56 ± 0.75	0.513
K2, postoperative (D)	43.83 ± 1.05	44.02 ± 1.21	0.349
Kmax, postoperative (D)	43.13 ± 0.82	43.32 ± 0.96	0.240
Anterior corneal astigmatism, postoperative (D)	0.9 (0.7–1.4)	1.5 (0.9–1.8)	**< 0.001**
Posterior corneal astigmatism, postoperative (D)	0.2 (0.2–0.3)	0.3 (0.2–0.3)	0.405

*SFIOL* Scleral fixation of intraocular lens, *IOL* Intraocular lens, *LogMAR* Logarithm of the minimum angle of resolution, *D* Diopter

^a^Difference between postoperative and preoperative measurements, negative values represent decrease in measurement. Data are summarized as mean ± standard deviation or median (1st quartile–3rd quartile) for continuous variables according to normality of distribution and as frequency (percentage) for categorical variables

Values that exhibit a statistically significant difference are highlighted in bold

## Discussion

Scleral fixation using the modified Yamane technique and the retropupillary iris-claw intraocular lens implantation approach have been shown to provide clinical outcomes comparable to those achieved with uneventful phacoemulsification combined with IOL implantation [2, 11, 12]. Among the two surgical methods recently utilized in aphakic patients, sutureless SFIOL is preferred for its minimal invasive advantage, while RPIOL implantation is favored for its ease of application and shorter surgical time advantages [5, 6]. Kelkar et al. found that the postoperative UCVA was 0.36 ± 0.32 logMAR in the RPIOL group and 0.30 ± 0.28 logMAR in the sutureless SFIOL group. They concluded that there was no significant difference between the two groups in terms of visual acuity at 1 year [10]. Madhivanan et al. reported that the BCVA at 1 year was 0.3 ± 0.2 with the sutureless SFIOL technique and 0.4 ± 0.4 with the RPIOL technique. They also found no significant difference between them [9]. Similar to our study, in both studies, the corneal incision was 2.8 mm in the sutureless SFIOL group, IOL were retroplaced in the RPIOL group, and IOL implantation was performed through a corneoscleral tunnel. The wound site was sutured with 10.0 nylon suture when necessary in these studies. In our case, a single continuous cross 10.0 nylon suture was placed in all cases. In this study, at the 1-year follow-up, statistically successful results were achieved in terms of UCVA and BCVA in both the sutureless SFIOL group and all patients in the RPIOL group, and there was no difference in vision between the two groups at the first year.

Various studies have shown increases in ACA, ACV, and ACD, as well as decreases in IOP after phacoemulsification. Additionally, studies have reported that lower levels of these parameters in the anterior segment often correspond to higher IOP values [7, 13, 14]. However, the effect of scleral fixation using the modified Yamane technique and the retropupillary iris-claw IOL implantation, which has recently become more common in aphakic patients, on the anterior segment, remains relatively unknown [15]. Yavuzer et al. stated that ACD, ACA, and ACV exhibited higher values in the sutureless SFIOL group compared to the phacoemulsification + IOL group. However, they reported a significant difference only in terms of ACV, while there was no significant difference in terms of ACD and ACA. Although not significant, they found lower IOP in sutureless SFIOL cases. They suggested that the higher ACD, ACA, and ACV values in this group may be attributed to the absence of capsular support behind the iris, and ACV may be more affected by the absence of capsular support [16]. In our study, no significant difference was found between the two groups in terms of ACD, ACV and ACA values. Although there are no studies evaluating ACD, ACV, and ACA in patients with RPIOL placement, the anterior segment values in the sutureless SFIOL group in our study are consistent with the literature. We attribute our finding of similar anterior segment values in both groups to the lack of posterior capsule support.

Madhivanan and colleagues found that the number of iritis cases and the frequency of IOP elevation (10%) were significantly higher in the iris-claw group compared to the sutureless SFIOL group. They suggested that this might be due to the absence of prophylactic iridectomy, and the pigment distribution caused by the enclavation of iris tissue [9]. In contrast, Kelkar et al. reported that the frequency of IOP elevation (17%) was higher in the sutureless SFIOL group compared to the iris-claw group [10]. Unlike these studies, which did not include prophylactic iridectomy, we performed prophylactic iridectomy in our iris-claw group. In our study, there was no significant difference between the two groups in terms of IOP. In our study, a statistically significant decrease in IOP was observed in both the sutureless SFIOL and the RPIOL group. No postoperative IOP elevation requiring anti-glaucomatous therapy was detected in any of the patients. There was a significant decrease in IOP after surgery in both groups compared to the preoperative period. We attribute this to the appropriately performed anterior vitrectomy in necessary patients and the fact that the IOL placed in a physiological position acts as a barrier for the vitreous.

It is crucial to implant the IOL as close as possible to the standard anatomical position to achieve the target refraction. Otherwise, it has been suggested that a 1 mm change in ACD corresponds to a 1.5 diopter change in refraction, leading to a myopic or hyperopic shift [17]. Yavuzer et al. found no significant difference between patients with sutureless SFIOL and those with phacoemulsification + IOL in terms of spherical values [16]. Madhivanan et al. stated that there was no statistically significant difference between the RPIOL and the sutureless SFIOL group in terms of spherical equivalent value [9]. In our study, no significant difference was detected between the two groups in terms of spherical values. This shows that the target refraction can be approached with IOL placed in normal physiological positions using appropriate calculation formulas.

It is recognized that corneal topographical values (K1 and K2) and corneal astigmatism can be influenced by the location and size of the corneal incision in phacoemulsification surgery [18]. In our study, IOL implantation with a 2.8 mm temporal incision was performed using the sutureless SFIOL method. Previous studies have indicated that with the 2.8 mm corneal incision in the sutureless SFIOL method, the values of K1, K2, and corneal astigmatism remained unchanged, as measured by the Scheimpflug corneal topography [19].

When Güell et al. compared patients who underwent RPIOL implantation in one eye and standard phacoemulsification + IOL implantation in the other eye, they found no significant difference in terms of BCVA and mean astigmatism [12]. In a study comparing two groups with sutured scleral fixation and RPIOL implantation, Kristianslund et al. reported that they did not detect a significant difference between the two groups in terms of postoperative cylindrical equivalent values and postoperative change in astigmatism [20]. In both studies, no corneal incision was made in any patient, and it was noted that low astigmatism was achieved with scleral incision and appropriate suturing. However, as a result of RPIOL implantation, an increase in K values and astigmatism can be observed depending on the location and size of the incision site [21, 22].

It has been demonstrated that a scleral tunnel incision in cataract surgery results in less astigmatism compared to a corneal incision of the same size [23]. In our study, we utilized a 5.5 mm scleral pocket arcuate incision for the RPIOL to minimize induced astigmatism. The incision site was sutured with crossed 10.0 nylon suture. When comparing the two groups 1 year after surgery, there was no significant change in K values and posterior astigmatism. Although anterior corneal astigmatism and cylindrical value were found to be higher in the RPIOL group, this difference did not impact the visual acuity of the two groups. Our study has several limitations, including its retrospective design and the absence of ultrasound microscopy or anterior segment OCT examinations to assess IOL status.

## Conclusion

In this study, statistically successful results were obtained in terms of UCVA and BCVA in the 1-year follow-up in the sutureless SFIOL and RPIOL groups. No difference was found in terms of spherical value, spherical equivalent, anterior chamber volume, anterior chamber depth, anterior chamber angle, K1, K2, Kmax, and posterior corneal astigmatism. In both surgical methods, there was a statistically significant decrease in intraocular pressure. Although there was a statistical difference in cylindrical value and anterior corneal astigmatism between the sutureless SFIOL and RPIOL groups, vision was not affected. According to this study, sutureless SFIOL and RPIOL are two successful methods in terms of visual acuity, anterior segment, and keratometry outcomes in aphakic patients after phacoemulsification. Long-term prospective studies with a larger number of patients are needed to determine the superiority of these methods over each other.
